# HLA alleles modulate EBV viral load in multiple sclerosis

**DOI:** 10.1186/s12967-018-1450-6

**Published:** 2018-03-27

**Authors:** Simone Agostini, Roberta Mancuso, Franca R. Guerini, Sandra D’Alfonso, Cristina Agliardi, Ambra Hernis, Milena Zanzottera, Nadia Barizzone, Maurizio A. Leone, Domenico Caputo, Marco Rovaris, Mario Clerici

**Affiliations:** 1 Don C. Gnocchi Foundation IRCCS – ONLUS, Piazzale Morandi 3, 20121 Milan, Italy; 2 Department of Health Sciences, University of Eastern Piedmont, Novara, Italy; 3 IRCCS Casa Sollievo della Sofferenza, San Giovanni Rotondo, Foggia Italy; 4 Department of Pathophysiology and Transplantation, University of Milan, Via Fratelli Cervi 93, 20090 Milan, Italy

**Keywords:** Epstein-Barr virus, HLA-class I alleles, HLA-A*02, HLA-B*07, Multiple sclerosis, Immunogenetics

## Abstract

**Background:**

The etiopathology of multiple sclerosis (MS) is believed to include genetic and environmental factors. Human leukocyte antigen (HLA) alleles, in particular,  are associated with disease susceptibility, whereas Epstein Barr Virus (EBV) infection has long been suspected to play a role in disease pathogenesis. The aim of the present study is to evaluate correlations between HLA alleles and EBV infection in MS.

**Methods:**

HLA alleles, EBV viral load (VL) and serum anti-EBV antibody titers were evaluated in EBV-seropositive MS patients (N = 117) and age- and sex-matched healthy controls (HC; N = 89).

**Results:**

Significantly higher DNA viral loads (p = 0.048) and EBNA-1 antibody titer (p = 0.0004) were seen in MS compared to HC. EBV VL was higher in HLA-B*07+ (p = 0.02) and HLA-DRB1*15+ (p = 0.02) MS patients, whereas it was lower in HLA-A*02+ (p = 0.04) subjects. EBV VL was highest in HLA-A*02−/B*07+/DRB1*15+ patients and lowest in HLA-A*A02+/B*07−/DRB1*15− individuals (p < 0.0001). HLA-B*07 resulted the most associated allele to EBV VL after multiple regression analysis considering altogether the three alleles, (p = 0.0001). No differences were observed in anti-EBV antibody titers in relationship with HLA distribution.

**Conclusions:**

Host HLA-B*07 allele influence EBV VL in MS. As HLA-class I molecules present antigens to T lymphocytes and initiate immune response against viruses, these results could support a role for EBV in MS.

**Electronic supplementary material:**

The online version of this article (10.1186/s12967-018-1450-6) contains supplementary material, which is available to authorized users.

## Background

Multiple sclerosis (MS), a chronic inflammatory disease of the central nervous system (CNS), is the most common cause of neurological disability in adults, and it affects over 2.5 million people worldwide [1]. The etiopathogenesis of the disease is still unclear and is believed to include both environmental and genetic factors, whose interplay leads to chronic activation of immune cells and neuronal damage. The major human leukocyte antigens (HLA) locus (6p21.3), in particular, was identified as the chromosomal region most strongly linked to MS [2], and a number of epidemiological studies identified HLA alleles that correlate with an increased risk of developing MS and that can influence disease progression in patients. Notably, the realization that different HLA alleles play a role in MS led to the understanding that this disease is, above all, an antigen-specific autoimmune disease [3]. To date, HLA-DRB1*15 is the strongest risk factor for the development of MS [4]. HLA-DRB1*15 is nevertheless not the only HLA allele related to MS: HLA-B*07+ patients are characterized by higher disease burdens and by a more evident degree of brain atrophy [5]. The presence of the HLA-A*02 allele, on the other hand, seems to be a protective factor against MS [6] and, in patients with the disease, it results in a more favorable clinical course, with a slower progression and a lower disease burden [7].

Amongst environmental factors, chronic viral infections, including those supported by Epstein Barr virus (EBV), are suspected to be involved in the initiation of MS [8]. EBV (also called human herpesvirus 4, HHV-4) is a ubiquitous human virus with a double stranded DNA genome (172 kbp long) wrapped in a protein capsid belonging to the *Herpesviridae* family, *Gammaherpesvirinae* subfamily, *Lymphocryptovirus* genus [9]. The virus is a worldwide pathogen and is persistently harbored by almost all adults regardless of health or geographic location. In western countries, with high standard of living, primary EBV infection occurs commonly in adolescence, and might cause infectious mononucleosis (IM) in a minority of cases. EBV replicates in oropharyngeal epithelial cells during primary disease and subsequently establishes a latent infection in circulating B lymphocytes.

IM and elevated titers of antibodies against EBV nuclear antigens (EBNA) have been associated to an increased risk to develop MS and, on the other hand, lack of EBV infection correlates with a lower risk to develop the disease [8]. Other epidemiologic results suggesting a role for EBV in the pathogenesis of MS include the observations that EBV-infected B cells and plasma cells accumulate in the brain of MS patients [10] and a strong EBV specific cell-mediated immune responses are seen in these patients [11, 12].

Moreover, evidences of EBV DNA or transcripts in brain or cerebrospinal fluid of MS individuals remains controversial [13], as reported by some papers [10, 14], but not confirmed by other authors [15–17].

Confliting results are reported on possible correlations between HLA alleles, EBV infection, and MS [18, 19]. Thus, an interaction between HLA-class I alleles and reactivity to EBV-related epitopes was recently shown, suggesting that the mechanism through which HLA genes influence the risk of developing MS may involve EBV-specific immune responses [18, 20]. Other data showed that HLA-B*7 allele is associated with increased EBV-specific Ab titers, higher disability scores, and a more compromised MRI pattern in MS patients [21]. We verified if correlations could be established between protective/risk HLA-class I alleles and EBV-specific parameters in MS; results suggest that this is indeed the case.

## Methods

### Study population and specimens

A total of 206 individuals were enrolled in the study: 117 of them (mean age: 44.7 ± 12.6 years) were patients with a diagnosis of MS according to revised McDonald criteria [22]; these patients are followed by the Multiple Sclerosis Unit of the Don C. Gnocchi Foundation, IRCCS S. Maria Nascente in Milan, Italy, and by the MS Centers of the PROGEMUS Consortium, coordinated at the University of Piemonte Orientale. The remaining 89 individuals (mean age: 44.8 ± 10.8 years) were age- and sex-matched healthy controls (HC), who donate blood at the same institutions. HC and MS patients were selected in order to obtain comparable distribution of HLA-B*07, -A*02 and -DRB1*15 frequency.

All individuals were EBV-seropositive. The study conformed to the ethical principles of the Declaration of Helsinki; all subjects gave informed and written consent according to a protocol approved by the local ethics committee of the centers.

Whole blood and serum samples were collected from all the individuals enrolled in the study. Whole blood was utilized for DNA extraction, whereas serum samples, obtained by centrifugation for 10 min at 1800*g* at room temperature, were aliquoted into sterile cryovials and stored at − 20 °C.

### DNA extraction

Genomic DNA was extracted from whole blood using standard phenol/chloroform procedure. DNA amount for each sample was determined by measuring the optical density at 260 nm wavelengths. DNA samples were stored at − 20 °C until use.

### HLA genotyping

The molecular genotyping of HLA-class I (HLA-A: 18 alleles and HLA-B: 38 alleles) and HLA-class II polymorphisms (HLA-DRB1: 18 alleles) was performed as previously described [23, 24].

### ELISA analysis

EBV seropositivity was measured in serum using commercial enzyme immunoassays (BEIA EBV EBNA-1 IgG Quant, Technogenetics, Milan, Italy, and EBV viral capsid antigen (VCA) IgG ELISA, IBL International, Hamburg, Germany), according to the manufacturer instructions.

EBNA-1-specific Ab (U/ml) were considered positive if > 180 U/ml, or negative if < 150 U/ml. Samples in grey zone (between 150 and 180 U/ml) were re-tested using the same immunoassay, and if repeatedly resulted in the grey zone, they was excluded from the analysis.

VCA-specific Ab (U/ml) were considered positive if > 22 U/ml, or negative if < 8 U/ml. Samples in grey zone (between 8 and 22 U/ml) were re-tested using the same immunoassay, and if repeatedly resulted in the grey zone, they was excluded from the analysis.

Cytomegalovirus (CMV) seropositivity was measured in serum using commercial enzyme immunoassay (CMV IgG ELISA, IBL International), according to the manufacturer instructions.

CMV IgG-specific Ab (U/ml) were considered positive if > 12 U/ml, or negative if < 8 U/ml. Samples in grey zone (between 8 and 12 U/ml) were re-tested using the same immunoassay, and if repeatedly resulted in the grey zone, they was excluded from the analysis.

### EBV and CMV DNA viral load

500 ng of genomic DNA was used as the template for quantitative evaluation by quantitative PCR (qPCR) using the ABI Prism 7000 sequence detection system (Applied Biosystems, Foster City, CA, US), and following a previously described protocol [25]. For EBV, the sequences of primers and probes were designed by Applied Biosystems on EBNA-LP region, whereas for CMV, primers CMVF (5′-CGCTCACATGCAAGAGTTAATCTTT), CMVR (5′AACTCGGTAAGTCTGTTGACATGTATG) and probe CMVP (FAM-CTCTATCTGACATACACAAGTAAATCCACGTCCCA-TAMRA) were selected from the UL123 gene using the Primer Express version 2.0.0 software. The EBNA-LP region is rich of variable copy numbers of a repeat domain: although the primers/probes were optimized by a commercial company, an effect on the results can’t be categorically exclude. EBV and CMV viral load was measured on a single occasion in each patient; each sample was tested in triplicate. Negative controls, as well as serial dilutions (from 10^4^ to 10 copies/μl for both EBV and CMV) of commercially available viral genomes (Advanced Biotechnologies Inc., Columbia, MD, USA), were included in each run. For both EBV and CMV the detection limit of this specific qPCR assay was 2.1 copies/μg DNA.

### Statistical analysis

Normally distributed data were summarized as mean and standard deviation (SD), or standard error mean (SEM), and comparison among groups were analyzed by ANOVA test and Student t test. Not-normally distributed data were summarized as median and interquartile range (IQR: 25th and 75th percentile), and comparisons were analyzed by Kruskal–Wallis test and Mann–Whitney U test, as appropriate. Multiple regression analysis was performed to verify the possible association between viral data and genetic parameters. Qualitative data were compared using Fisher’s exact test.

## Results

### Clinical characterization of study subjects

Clinical characteristics of the individuals enrolled in the study are shown in Table 1. Demographic analysis showed that age and sex were not different when MS patients and HC were compared. Among MS patients, 77 were affected by relapsing–remitting disease (RR-MS) in a phase of disease stability, whereas 40 had a diagnosis of progressive MS (P-MS, 15 with primary form and 25 with secondary form). As per definition, the expanded disability status scale (EDSS) score was significantly higher in P-MS (6.1 ± 2.1) than in RR-MS (2.9 ± 1.6, p < 0.0001). Within the MS group of patients the age at disease onset was significantly lower in RR-MS compared to P-MS (p = 0.04), but the disease duration was comparable (data not shown).

**Table 1:** Demographic, clinical and genetic characteristics of the individuals enrolled in the study

	Healthy controls (HC)	Multiple sclerosis patients (MS)	Relapsing–remitting MS patients (RR-MS)	Progressive MS patients (P-MS)
N	89	117	77	40
Gender (M:F)	43:46	48:69	32:45	16:24
Age (years)	44.8 ± 10.8	44.7 ± 12.6	42.1 ± 9.5	50.8 ± 10.2
EDSS	–	3.8 ± 2.2	2.9 ± 1.6	6.1 ± 2.1*
HLA-A*02 carriers (%)	64	62	66	60
HLA-B*07 carriers (%)	69	60	61	57
HLA-DRB1*15 carriers (%)	68	64	63	68

Data are expressed as mean ± standard deviation

*EDSS* Expanded Disability Status Scale

* p < 0.0001 vs. RR-MS

### EBV seropositivity and viral load

All the 206 individuals included in the study were IgG EBNA-1 and VCA seropositive. The EBNA-1 antibody titer was significantly higher in MS (1009.23; 782.79–1383.37 U/ml) compared to HC (782.55; 627.82–910.95 U/ml; p < 0.0001). This difference remained significant when MS patients were split in RR-MS (p = 0.0008 vs. HC) and in P-MS (p = 0.03 vs. HC). IgG EBNA-1 Ab titers were comparable in RR-MS and P-MS patients (Table 2).

**Table 2:** EBV and CMV seroprevalence and antibodies titers in MS patients and controls

	Healthy controls (HC)	Multiple sclerosis patients (MS)	Relapsing–remitting MS patients (RR-MS)	Progressive MS patients (P-MS)
EBV seroprevalence (%)	100	100	100	100
EBNA-1 IgG (U/ml)	782.55 (627.82–910.95)	1009.23* (782.79–1383.37)	1139.00* (782.70–1452.15)	937.29** (828.82–1191.21)
VCA Ab (U/ml)	375.54 (144.77–625.19)	969.07** (511.62–1340.41)	1146.45** (560.95–1407.95)	885.15 (277.02–1050.24)
CMV seroprevalence (%)	64.3	70.2	69.7	71.2
CMV IgG (U/ml)	58.40 (38.15–84.47)	83.40 (46.33–119.56)	80.87 (46.78–124.58)	86.68 (55.04–112.95)

Data are expressed as medians and (interquartile range)

*EBV* Epstein-Barr Virus, *CMV* cytomegalovirus, *EBNA1* EBV nuclear antigen 1, *VCA* viral capsid antigen, *IgG* immunoglobulin G, *VL* viral load

* p < 0.0001 vs. HC

** p < 0.05 vs. HC

The VCA antibody titer was significantly higher in MS (969.07; 511.62–1340.41 U/ml) compared to HC (375.54; 144.77–625.19 U/ml; p = 0.02). When MS patients were split in RR-MS and in P-MS, this difference remained significant between RR-MS and HC (p = 0.01), whereas no significant differences were observed between P-MS and HC, and between RR-MS and P-MS (Table 2).

**Fig. 1:**
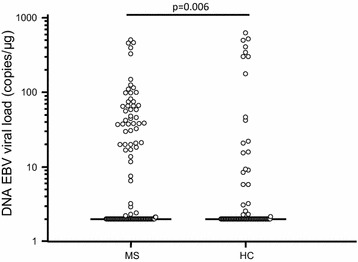
EBV viral load in blood of multiple sclerosis patients and healthy controls

EBV DNA was significantly more frequently detected in MS patients (54/117; 46.1%) compared to HC (25/89; 28.1%) (p = 0.009). Moreover, although the median is the same in MS and HC group (2.0 copies/μg), the overall EBV DNA viral load is significantly increased in MS patients (IQR: 2.00–37.50 copies/μg) than HC (IQR: 2.00–2.36 copies/μg) (p = 0.006) (Fig. 1).

Finally, no differences were detected when either EBV DNA or viral loads were compared between RR-MS (EBV DNA positivity: 48%; EBV viral load: 2.0; 2.0–39.3 copies/μg) and P-MS (EBV DNA positivity: 43%; EBV viral load: 2.0; 2.0–28.9 copies/μg) patients. As previously indicated, all the individuals enrolled in the study were EBV seropositive; some of them, nevertheless, had an undetectable EBV viral load. An arbitrary value < 2 copies/μg (i.e. the limit detection of the assay) was assigned to these samples for the analysis of association with HLA genotyping. No correlation was observed between EBV viral load and EBV or VCA Ab titers, either considering the enrolled subject altogether, either considering the enrolled individuals as a group or splitting them in MS and HC (see Additional file 1: Figure S1).

### CMV seropositivity and viral load

The 70.2% of MS and the 64.3% of HC were CMV IgG seropositive. The antibody titer was similar in the two groups (MS: 83.40; 46.33–119.56 U/ml; HC: 58.40; 38.15–84.47 U/ml) (Table 2), and no differences were observed also splitting the patients in RR-MS and P-MS. The CMV viral load was not detectable in blood in all the enrolled subjects but in one healthy control (VL: 65.75 copies/μg).

### HLA genotyping and EBV

**Fig. 2:**
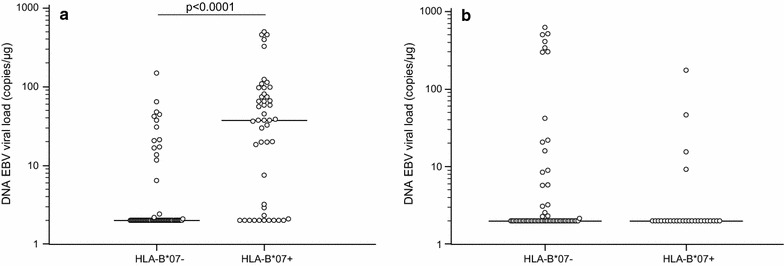
EBV viral load in blood of multiple sclerosis patients (**a**) and healthy controls (**b**) carrying HLA-B*07 allele

**Fig. 3:**
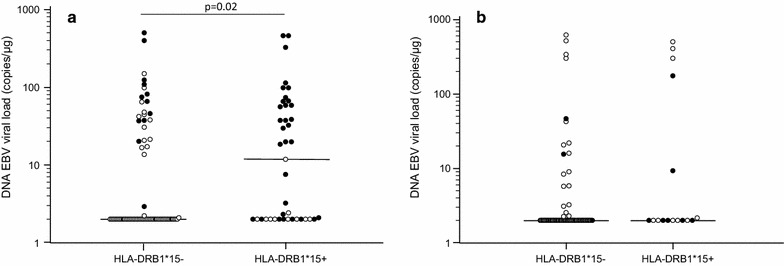
EBV viral load in blood of multiple sclerosis patients (**a**) and healthy controls (**b**) carrying HLA-DRB1*15 (**c**) allele

HLA-A, -B, and -DRB1 genotyping was performed in all the enrolled subjects and genetic patterns were evaluated in relationship with EBV parameters. The HLA frequencies are summarized in Table 1. Results showed that a significantly higher EBV viral load could be detected in MS subjects carrying the HLA-B*07 allele (non-protective allele) (HLA-B*07+) (N = 47/117) (37.54; 2.0–74.25 copies/µg) compared to those not carrying this allele (HLA-B*07−) (N = 70/117) (2.0; 2.0–2.0 copies/μg; p < 0.0001) (Fig. 2a). Similarly, MS patients carrying the HLA-DRB1*15 allele (risk allele) (HLA-DRB1*15+) (N = 42/117) had higher EBV viral load (11.7; 2.0–58.3 copies/µg) than those not carrying this allele (HLA-DRB1*15−) (N = 75/117) (2.0; 2.0–21.1 copies/µg) (Fig. 3a) (p = 0.02).

**Fig. 4:**
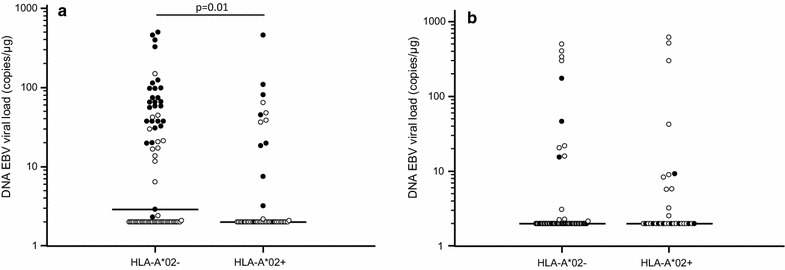
EBV viral load in blood of multiple sclerosis patients (**a**) and healthy controls (**b**) carrying HLA-A*02. Black dots represent subjects carrying HLA-B*07 allele

**Fig. 5:**
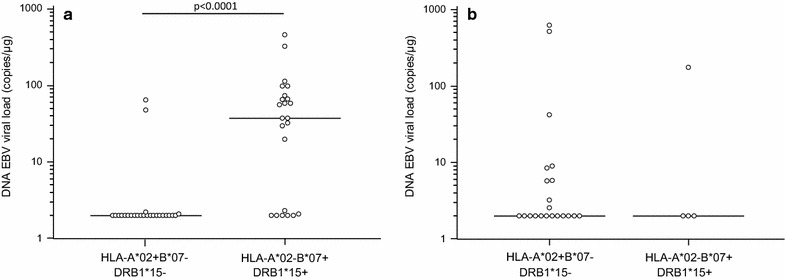
EBV viral load in blood of multiple sclerosis patients (**a**) and of healthy controls (**b**) in relationship with the co-segregation of HLA-A*02, -B*07 and -DRB1*15 alleles

Conversely, a significantly lower EBV viral load was observed in MS patients expressing the HLA-A*02 antigen (HLA-A*02+) (N = 44/117) (2.0; 2.0–2.1 copies/µg), which is suggested to be a protective factor in MS, compared to those without the allele (HLA-A*02−) (N = 73/117) (2.9; 2.00–37.65 copies/µg; p = 0.01) (Fig. 4a). EBV viral load was next evaluated in MS patients in relationship with the different distribution pattern of HLA-A*02, -B*07 and -DRB1*15 alleles. Results showed that the highest EBV viral load (37.5; 2.1–72.1 copies/µg) was detected in HLA-B*07+/HLA-DRB1*15+/HLA-A*02− (least favorable HLA combination) patients (N = 23); conversely, the 25 HLA-B*07−/HLA-DRB1*15−/HLA-A*A02+ patients were characterized by the lowest EBV viral load (2.0; 2.0–2.0 copies/µg) (p < 0.0001) (Fig. 5a).

Moreover, a multiple regression analysis where EBV viral load was computed as dependent variable and HLA-B*07, -A*02 and –DRB1*15 alleles as independent variables, confirmed only the association of HLA-B*07 with high EBV viral load (p = 0.0001). These data suggest that the risk effects of the HLA-B*07 allele are stronger than the “protective” ones attributed to the HLA-A*02 allele and than the “risk” ones attributed to the HLA-DRB1*15 allele. Notably, the relationship between HLA alleles and viral parameters was exclusively seen in MS patient as no such effects could be observed in the 89 HC (Figs. 2, 3, 4 and 5b).

**Fig. 6:**
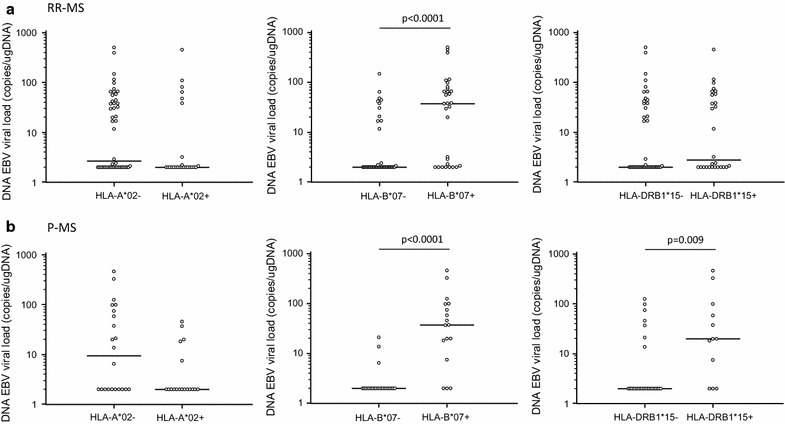
EBV viral load in blood of RR-MS (**a**) and P-MS (**b**) carrying HLA-A*02, -B*07 and -DRB1*15 alleles

Splitting MS patients between RR-MS and P-MS, results regarding the association between EBV viral load was significantly higher in RR-MS expressing the HLA-B*07 antigen (37.5; 2.1–74.0 copies/µg) than those not carrying this allele (2.0; 2.0–4.7 copies/µg; p < 0.0001). EBV viral load was significantly increased as well in P-MS carrying -B*07 (37.5; 15.6–97.79 copies/µg) compared to P-MS patients who did not carry this allele (2.0; 2.0–2.0 copies/µg; p < 0.0001). Regarding the others alleles, results were confirmed for DRB1*15 in P-MS patients (p = 0.009) (Fig. 6). Notably, comparable EBV viral load characterized RR-MS and P-MS expressing the same HLA molecules.

Finally, no relationships were found between HLA distribution and EBV-specific antibody titers, either considering the enrolled individuals as a group or splitting them in MS and HC.

## Discussion

The etiology of MS is still unknown, but it is widely accepted that interactions between environmental and genetic factors shape the susceptibility to this multifactorial disease. Thus, whereas the correlation between certain HLA alleles and disease susceptibility has been definitely established, the possible association between EBV infection and MS has been repeatedly suggested but is still in need of convincing and definitive conclusions.

Herein we initially analyzed EBV infection parameters in a MS study population; our results confirmed previous data indicating that higher anti-EBNA-1 IgG serum titers are present in MS patients compared to HC [8, 26]. We also observed that, in MS patients compared to controls: (1) EBV DNA in blood is more frequently detected, and (2) EBV viral loads are significantly increased, although these viral loads are not comparable to those seen in EBV-associated malignancies [27]. These data confirm previously published results [28, 29], even if not all authors confirm such findings [30, 31], and could be the consequence of increased rates of EBV replication, more frequent reactivations, or of higher frequencies of circulating EBV-infected B lymphocytes [32]. However, it is important to note that, because in our experiments DNA was extracted from whole blood, viral DNA originating from B cells could not be discriminated from that originating from virions.

No relations were observed between EBV-specific antibody titers and EBV viral load in the study population. These results are partially in agreement with recent results indicating that, whereas VCA-specific Ab titers do correlate with EBV viral load, EBNA-1-specific Ab do not [33]; analyses on wider cohorts of individuals will be needed to clarify this aspect.

Having covered the virological side, we next analyzed whether polymorphisms in the HLA region known to modulate MS susceptibility [34] and its progression could associate with virologic parameters. Several lines of evidence indicate the presence of possible interactions between EBV infection-associated parameters, usually EBNA-1 antibody titers, and HLA profiles in MS; these evidences are nevertheless not unequivocal [18–21, 35, 36]. Whereas in our cohort we did not observe any correlations between anti-EBV antibody titers and HLA antigens, possibly because of the small number of individuals analyzed, clear correlations emerged between HLA alleles and EBV viral loads. Thus, results showed that patients carrying the MS risk allele B*07 (HLA-B*07+) in the presence of the risk allele DRB1*15 and in the absence of the protective allele A*02 (HLA-B*07+/DRB1*15+/A*02−) were characterized by the presence of the highest EBV viral loads. These findings were confirmed by the observation that the lowest EBV viral loads were seen in MS patients showing the opposite HLA profile (HLA-B*07−/DRB1*15−/A*02+). The same trend remained when HLA-B*07, DRB1*15 and HLA-A*02 alleles were analyzed singularly. To note, analyzing the effects of HLA alleles altogether by multiple regression analysis, HLA-B*07 allele appears to have the strongest role on EBV DNA viral load. It is noteworthy that HLA-B*07 belongs to the same HLA haplotype carrying the HLA-DRB1*15 allele, whose association with MS susceptibility is well-established. Recently, a role of B*07 in EBV infection in MS patients has been proposed [12]. It is also important to observe that these correlations disappeared when the analyses were performed in healthy controls.

## Conclusions

Merging the virologic and the genetic results presented herein allows the speculation that the increased blood viral loads seen in our patients are the consequence of a suboptimal control of EBV replication/reactivation by HLA-B*07+ restricted CD8^+^ T cell. Notably, HLA-B*07 antigen was recently shown to only weakly present viral epitopes to cytotoxic T lymphocytes [11]. This would allow more frequent EBV lytic replication/reactivation and the infection of an ampler pool of naïve B lymphocytes.

The suboptimal control of EBV replication/reactivation observed in HLA-B*07+ MS subjects may have had an impact on disease severity, but interestingly, no differences in EBV viral load were observed between HLA-B*07+ restricted RR- and P-MS patients. Finally, it could be expected that the frequency and/or the potency of EBV-specific cytotoxic T cell would be different in MS patients expressing (or lacking) the different HLA alleles analyzed in this study. We are currently enrolling patients and controls selected according to their HLA profiles to verify this hypothesis. Interestingly, these results are in line with several analyses performed in other diseases in which the etiologic role of EBV is suspected, or has been definitely confirmed. Thus, the presence of the HLA-A*02 allele was shown to be protective in nasopharyngeal carcinoma and in Hodgkin’s lymphomas [37, 38]. Taken together results herein reinforce the hypothesis that the role of HLA-class I antigens in the pathogenesis of MS could be at least partially mediated by their ability to control EBV infection and persistence.

## Additional file

**Additional file 1: Figure S1.**Correlation between DNA EBV viral load and EBV Ab titers in study population. Correlation between DNA EBV viral load and Ab EBNA-1 titers (A) or Ab VCA titers (B) in study population.

## Abbreviations

Ab: antibody
ANOVA: analysis of variance
CMV: cytomegalovirus
CNS: central nervous system
DNA: deoxyribonucleic acid
EBNA-1: EBV nuclear antigens
EBV: Epstein Barr-virus
EDSS: Expanded Disability Status Scale
HC: healthy controls
HHV-4: human herpesvirus 4
HLA: human leukocyte antigen
IgG: immunoglobulin G
IM: infectious mononucleosis
IQR: interquartile range
MRI: magnetic resonance imaging
MS: multiple sclerosis
P-MS: progressive MS
RR-MS: relapsing-remitting MS
SD: standard deviation
SEM: standard error mean
VCA: viral capsid antigen
VL: viral load
