# Supplementary material for: HLA alleles modulate EBV viral load in multiple sclerosis
Source: J Transl Med. 2018 Mar 27;16:80. doi: 10.1186/s12967-018-1450-6 (PMC5870171; doi:10.1186/s12967-018-1450-6)
Supplement: Supplementary file 1 — Additional file 1: Figure S1.Correlation between DNA EBV viral load and EBV Ab titers in study population. Correlation between DNA EBV viral load and Ab EBNA-1 titers (A) or Ab VCA titers (B) in study population. [file 12967_2018_1450_MOESM1_ESM.docx]

**HLA alleles modulate EBV viral load in Multiple Sclerosis**

Simone Agostini^1^, Roberta Mancuso^1^, Franca R. Guerini^1^, Sandra D’Alfonso^2^, Cristina Agliardi^1^, Ambra Hernis^1^, Milena Zanzottera^1^, Nadia Barizzone^2^, Maurizio A. Leone^3^, Domenico Caputo^1^, Marco Rovaris^1^, Mario Clerici^1,4^.

^1^Don C. Gnocchi Foundation IRCCS – ONLUS, Piazzale Morandi 3, 20121, Milano, Italy

^2^Department of Health Sciences, University of Eastern Piedmont, Novara, Italy

^3^IRCCS Casa Sollievo della Sofferenza, San Giovanni Rotondo, Foggia, Italy

^4^Department of Pathophysiology and Transplantation, University of Milan, Via Fratelli Cervi 93, 20090, Milano, Italy

**Additional file**

**Figure 1S: Correlation between DNA EBV viral load and EBV Ab titers in study population.** Correlation between DNA EBV viral load and Ab EBNA-1 titers (panel A) or Ab VCA titers (panel B) in study population.
